# MYB Superfamily in *Brassica napus*: Evidence for Hormone-Mediated Expression Profiles, Large Expansion, and Functions in Root Hair Development

**DOI:** 10.3390/biom10060875

**Published:** 2020-06-07

**Authors:** Pengfeng Li, Jing Wen, Ping Chen, Pengcheng Guo, Yunzhuo Ke, Mangmang Wang, Mingming Liu, Lam-Son Phan Tran, Jiana Li, Hai Du

**Affiliations:** 1 College of Agronomy and Biotechnology, Chongqing Engineering Research Center for Rapeseed, Southwest University, Chongqing 400716, China; pengfengli17@126.com (P.L.); luckywenjing@aliyun.com (J.W.); chenp1996@126.com (P.C.); pengcheng0813@aliyun.com (P.G.); kyz2014@email.swu.edu.cn (Y.K.); mangmangwang16@126.com (M.W.); lmm_xqzr@163.com (M.L.); ljn1950@swu.edu.cn (J.L.); 2 Academy of Agricultural Sciences, Southwest University, Chongqing 400716, China; 3 Institute of Research and Development, Duy Tan University, 03 Quang Trung, Da Nang 550000, Vietnam

**Keywords:** *Brassica napus*, MYB transcription factor, complementation, evolution, expression, root hair development

## Abstract

MYB proteins are involved in diverse important biological processes in plants. Herein, we obtained the *MYB* superfamily from the allotetraploid *Brassica napus*, which contains 227 *MYB*-related (*BnMYBR*/*Bn1R-MYB*), 429 *R2R3-MYB* (*Bn2R-MYB*), 22 *R1R2R3-MYB* (*Bn3R-MYB*), and two *R1R2R2R1/2-MYB* (*Bn4R-MYB*) genes. Phylogenetic analysis classified the Bn2R-MYBs into 43 subfamilies, and the BnMYBRs into five subfamilies. Sequence characteristics and exon/intron structures within each subfamily of the *Bn2R-MYB*s and *BnMYBR*s were highly conserved. The whole superfamily was unevenly distributed on 19 chromosomes and underwent unbalanced expansion in *B. napus*. Allopolyploidy between *B. oleracea* and *B. rapa* mainly contributed to the expansion in their descendent *B. napus*, in which *B. rapa*-derived genes were more retained. Comparative phylogenetic analysis of 2R-MYB proteins from nine Brassicaceae and seven non-Brassicaceae species identified five Brassicaceae-specific subfamilies and five subfamilies that are lacking from the examined Brassicaceae species, which provided an example for the adaptive evolution of the 2R-MYB gene family alongside angiosperm diversification. Ectopic expression of four *Bn2R-MYB*s under the control of the viral *CaMV35S* and/or native promoters could rescue the lesser root hair phenotype of the *Arabidopsis thaliana wer* mutant plants, proving the conserved negative roles of the *2R-MYB*s of the S15 subfamily in root hair development. RNA-sequencing data revealed that the *Bn2R-MYB*s and *BnMYBR*s had diverse transcript profiles in roots in response to the treatments with various hormones. Our findings provide valuable information for further functional characterizations of *B. napus* *MYB* genes.

## 1. Introduction

MYB proteins are characterized by a conserved DNA-binding domain comprising 1–4 conserved MYB repeats (R1–R3) [1,2,3]. MYB proteins are widely distributed in eukaryotes with many more homologs in plants, which comprise a transcription factor (TF) gene superfamily in plant genomes [4,5]. This superfamily has been generally categorized into four major MYB-type families: 2R-MYB (R2R3-MYB), 3R-MYB (R1R2R3-MYB), 4R-MYB (R1R2R2R1/2-MYB), and MYB-related (MYBR/1R-MYB) families, based on the number of their MYB repeats [3]. The MYBR and 2R-MYB proteins form the largest families that usually have from dozens to hundreds of members in higher plants, whereas the other two families have much lower numbers of members and generally have only several members in some species [4,5].

The *2R-MYB* genes encode the most typical plant MYB proteins. Since the first *2R-MYB* gene was found to be implicated in the maize anthocyanin biosynthesis [1], the *2R-MYB* family has been detected in a much wider range of plant species, and represents a large TF family in plant genomes [5,6]. Thus, a great number of recent research has focused more intensively on the roles of *2R-MYB* genes in evolution [3,5,6], and the regulation of different biological processes including secondary metabolism [3,7,8], plant development (e.g., root hair development) [9,10] as well as plant responses to environmental stresses [11,12] and different types of hormones [13,14]. The MYBR proteins have also been demonstrated to play key roles in many important biochemical, developmental, and physiological processes in plants such as regulating circadian rhythms [15], anthocyanin biosynthesis [16], trichome and root hair development [17], and triacylglycerol accumulation [18]. The plant 3R-MYB proteins are homologs of animal MYB proteins [19], which play a conserved role in cell cycle regulation [19,20] as well as plant abiotic stress responses [21]. However, the functions of *4R-MYB* genes remain unclear.

Genome-wide analysis of the *MYB* superfamily has been carried out in many plant species including *Arabidopsis thaliana* [22], *Oryza sativa* (rice) [23], and *Glycine max* (soybean) [6], etc. Most of these studies focused on the typical *2R-MYB* family, and only a few works have addressed the whole superfamily [6,24,25]. Previously, we carried out a global analysis of the *2R-MYB* families across 50 representative eukaryotes and provided a systemic classification for this family [5]. Similarly, we also assessed the *MYBR* families in 16 major land plants [4]. We found that these two families rapidly expanded alongside the diversification of higher plants, forming a range of new functions, with the *2R-MYB*s forming many ’orphan’ genes and/or species-specific subfamilies or clades during evolution [4,5]. However, a detailed evolutionary history of the *MYB* superfamily in many plant genomes has not yet been elucidated by any study, since the *MYB* superfamily has a huge number, from hundreds to thousands, of members. Furthermore, detailed mechanisms underlying gene expansion and the subsequent evolution of plant *MYB* duplicates also remain unknown. Gaining deeper insights into the evolutionary history of all the *MYB* members in a specific evolutionary lineage of plants may have implications for an in-depth understanding of the evolution and classification of the *MYB* superfamily.

Brassicaceae is a large eudicot family that includes *A. thaliana* and many important crops for human food. This family represents one of the largest lineages with a mass of available sequenced genomes; and thus, may have potential for further research on genome duplication and evolution. In the present study, we identified and classified the *MYB* superfamily in allotetraploid *Brassica napus* (genome A_n_A_n_C_n_C_n_), and its ancestors *B. rapa* (genome ArAr) and *B. oleracea* (genome CoCo) at a genome-wide level. We focused on exploring the evolutionary history of this superfamily after the recent allopolyploidy event between *B. oleracea* and *B. rapa*. Furthermore, given an evident boosting trend of new subfamilies in the *2R-MYB* family, to gain further insights into the evolutionary mechanisms of this gene family, we conducted a phylogenetic analysis of all putative *2R-MYB* genes identified in 16 species (including nine Brassicaceae and seven closely related non-Brassicaceae species). These data allowed us to provide a more detailed classification and assessment of the evolutionary patterns of differentiation and proliferation of the *2R-MYB* family in higher plants. As a means to study the function of several representative members of the *Bn2R-MYB* subfamily, on the basis of homology with the *A. thaliana MYB066* (*WER*) gene, we selected four *2R-MYB* genes for their functional analysis. Our results revealed that these four selected genes could rescue the aberrance in root hair development of the *A. thaliana wer* mutant plants, indicating a positive correlation between sequence homology and biological function. Finally, to identify the *BnMYB* genes, which act in the roots in a hormone-dependent manner, we analyzed the RNA-sequencing (RNA-seq) data of the roots of *B. napus MYB* plants exposed to various hormone treatments.

## 2. Materials and Methods

### 2.1. Sequence Retrieval of MYB Genes

To identify the MYB proteins in *Brassica napus*, BLASTP was applied to search against the proteome of *B. napus* (Darmor–*bzh* ecotype) available in GENOSCOPE [26]. To ensure that no MYB proteins were eliminated by lack of correspondence to the consensus, a representative sequence from each subfamily of the MYBR, 2R-MYB, 3R-MYB, and 4R-MYB proteins [4,5,22] was used as a query in the BLASTP search with a low-stringency stringent criterion (cutoff *P* < 0.1). Each matching sequence was subsequently used to search in the *B. napus* genome database until no new sequence was found. After removing the incomplete or redundant sequences, putative MYB protein sequences were further confirmed using the PROSITE database [27] and the SMART database [28] to confirm that the candidates possessed the typical MYB domain. The identified sequences were then considered as candidates based on the following three additional criteria: (i) the MYB domain had a significant sequence similarity to that of typical plant MYB proteins; (ii) each MYB repeat contained at least two of the three highly conserved tryptophan (W) residues; and (iii) MYB repeats were adjacent to each other [4,5]. To ensure the integrity of the data, among the sequences that possess incomplete open reading frames (ORFs), only those that have a long deletion in the MYB domain, which will impact the phylogenetic analyses (no common sites for the sequence pair), were excluded from further analyses. Finally, the positive hits were classified into four distinct MYB-types (i.e., MYBR-, 2R-MYB-, 3R-MYB- and 4R-MYB-types according to the detection of one, two, three, and four MYB repeats, respectively). Meanwhile, we also identified the *MYB* genes from another sequenced *B. napus* (cv. ZS11) genome found in the NCBI database [29], and compared the sequences of candidates identified from these two *B. napus* cultivars using the MEGA v5.2 [30]. All the newly identified sequences were then named according to their chromosomal location. The corresponding cDNA and genomic sequences of identified candidates were also acquired from these two genomes.

To further explore the expansion of *2R-MYB* genes across the Brassicaceae family or closely related species, the *2R-MYB*s in *B. rapa FPsc* v1.3*, B. oleracea* v1.0, *Boechera stricta* v1.2, *Capsella grandiflora* v1.1, *Eutrema salsugineum* v1.0, *Citrus clementina* v1.0, *Citrus sinensis* v1.1, *Theobroma cacao* v1.1, *Gossypium raimondii* v2.1, *Carica papaya ASGPB* v0.4, *Capsella rubella* v1.0, *A. lyrata* v1.0, *Manihot esculenta* v6.1, and *Eucalyptus grandis* v2.0 were identified from Phytozome v12.1 [31] using the same method. The newly identified candidate sequences for each Brassicaceae species along with the previously reported *A. thaliana* sequences [22] and the candidates identified in *B. napus* (Darmor–*bzh* ecotype) (this work) were collected into a preliminary dataset for further analyses.

### 2.2. Sequence and Phylogenetic Analyses

The intron/exon structures and intron phases of *B. napus MYB* genes (*BnMYB*s) were analyzed by aligning the genomic and coding sequence (CDS) of each gene using the MUSCLE software in MEGA v5.2 [30] with the default parameters. The gene structures of *BnMYB*s were then confirmed and visualized with Gene Structure Display Server 2.0 with the default parameters [32]. Subsequently, the intron patterns (including their distribution, positions and phases) of each gene were determined based on the presence of intron(s) in the genomic region spanning the MYB domain encoded by each *MYB* gene. This region was only used in intron pattern analysis because the length and sequence homology of the other regions were quite divergent. The physical and chemical characteristics (e.g., number of amino acids, theoretical isoelectric points (pI) and molecular weight) of each candidate protein were predicted using the Protparam tool [33] with the default parameters. Subcellular localization prediction was performed by the Plant-mPLoc online tool with the default parameters [34].

Multiple sequence alignment of the protein sequences of the MYB domains was carried out using the online MAFFT v7 under the default parameters [35], and the aligned sequences were then manually checked using the MEGA v5.2 [30]. An unrooted phylogenetic tree was constructed using the neighbor-joining (NJ) method in MEGA v5.2 with 1000 replicates based on proportion distance (p-distance) and pairwise deletion. The tree file was viewed and edited using the FigTree v1.3.1 (http://tree.bio.ed.ac.uk/ software/figtree).

### 2.3. Chromosomal Locations and Collinearity of MYB Genes in B. napus

The chromosomal distribution of putative *MYB* genes in the *B. napus* genome was viewed in MapChart. Collinearity analysis of *A. thaliana*, *B. oleracea*, *B. rapa*, and *B. napus* genomes was carried out in CoGe (https://genomevolution.org/CoGe) [36]. The colinear relationships of putative *MYB* genes were identified based on the cross-genome collinearity analysis, and the duplication events were accordingly defined as follows: (i) homeologous exchanges (HE): transfer of genetic information between homeologous sequences of different subgenomes; (ii) segmental exchanges (SE): transfer of genetic information between different chromosomal sequences; (iii) segmental duplications (SD): duplicated copies of chromosomal sequences; (iv) tandem duplications (TD): closely related *MYB* genes in a single cluster in the phylogenetic tree, which were physically located close to each other on a given chromosome without intervening sequences. The HE, SE, and SD events were distinguished from each other based on the chromosomal homology and the colinear relationship (orthologous gene pairs in orthologous blocks) of the A_n_ (derived from *B. rapa*) and C_n_ (derived from *B. oleracea*) subgenomes and their respective progenitor genomes (*B. rapa* and *B. oleracea*) in all possible combination pairs. The intron/exon organization and chromosomal information of putative *MYB* genes in other species were acquired from the Phytozome v12.1 or BRAD database [37].

### 2.4. Spatial and Hormone-Induced Expression of B. napus MYB Genes

The expression profiles of *MYB* candidates in the roots of *B. napus* seedlings (five-leaf stage) exposed to auxin (IAA), gibberellin (GA_3_), cytokinin (6-BA), abscisic acid (ABA), and ethylene (ACC) treatments, respectively, were evaluated using the RNA-seq data recently created by our lab (BioProject ID PRJNA608211). Briefly, seeds of the ZS11 cultivar were obtained from the College of Agriculture and Biotechnology, Southwest University (Chongqing, China) and grown in soil (nutrition soil : rock = 2:1, v:v) in a plant incubator under the long-day conditions (day/night = 16/8 h). Seedlings with four expanded leaves (four-leaf stage) were transferred to Hoagland liquid medium, and grown in an artificial climate chamber at 25 °C under a 16/8 h (day/night) photoperiod. The seedlings at the five-leaf stage were then treated in Hoagland solution containing indicated concentrations of phytohormones (50 µM ABA, 120 µM GA_3_, 75 µM 6-BA, 60 µM ACC, and 10 µM IAA, respectively). Root samples were then harvested at 0, 1, 3, 6, 12, and 24 h after the treatments, and were immediately frozen in liquid nitrogen and stored at −80 °C until RNA isolation. RNA-Seq was performed using the Illumina sequencing platform (HiSeq 2000) at Biomarker Technologies Co. Ltd. (Beijing, China) (http://www.biomarker.com.cn).

Genes with no or low expression levels (fragments per kilobase of transcript per million mapped reads (FPKM) < 1) in all of the 60 samples might represent pseudogenes or might be expressed only under special condition(s) or at specific developmental stage(s); thus, they were removed from further analyses. The RNA-Seq data of *MYB* candidate genes were log_2_-transformed and median-centered. Then, hierarchical clustering of log_2_-transformed RNA-Seq data was analyzed using Cluster 3.0 [38]. The heatmaps were viewed in Java Treeview [39].

### 2.5. Cloning of B. napus WER-Homologous Genes

The full-length CDSs of four *B. napus 2R-MYB* genes (*BnMYB019*, *BnMYB189*, *BnMYB231*, and *BnMYB388*) that showed high homology to the *A. thaliana MYB066* (*WER*) [10] were polymerase chain reaction (PCR)-amplified using the cDNA sample prepared from roots with gene-specific primers (Table S1). The PCR reaction was carried out using *kodakarensis* (KOD) plus DNA polymerase (ToYoBo, Shanghai, China) in a total volume of 50 μL. The procedure was as follows: an initial denaturing step at 94 °C for 5 min; 34 cycles of 94 °C for 30 s, 55 °C for 30 s, and 68 °C for 50 s; and a final extension step at 68 °C for 10 min. Similarly, the 1.5-kb promoter sequences of the *BnMYB019* and *BnMYB231* genes were amplified from the *B. napus* genome by PCR using the PrimeSTAR HS DNA Polymerase (TakaRa, Dalian, China).

### 2.6. Plasmid Construction and Transformation

The corresponding CDSs of the four selected *B. napus 2R-MYB* genes were first cloned into the binary vector pRI201 (TakaRa), and then subcloned into the pCAMBIA1301 vector with the *Pst*I and *Sma*I sites for ectopic expression in *A. thaliana*. The resulting vectors (*35S::BnMYB019*, *35S::BnMYB189*, *35S::BnMYB231*, and *35S::BnMYB388*) contained the selected genes under the control of the cauliflower mosaic virus (CaMV) *35S* promoter, and the *phosphinothricin* gene as a plant-selectable marker conferring Basta resistance. Similarly, the native promoters of *BnMYB019* and *BnMYB231* were digested with *Pst*I and *Xba*I, and were used to replace the *35S* promoter in the *35S::BnMYB019* and *35S::BnMYB231* plasmids, resulting in the *BnMYB019::BnMYB019* and *BnMYB231::BnMYB231* constructs, respectively, for the functional complementation of the *A. thaliana wer* mutant plants. All of the constructs were sequenced to check for PCR-induced errors.

Gene constructs were transferred into *Agrobacterium tumefaciens* GV3101 by the freeze–thaw method. *A. thaliana* plants (wild-type (WT) and *wer* mutant; both are Col-0 ecotype) were transformed with the GV3101 transformants by the floral dipping method, and transgenic plants were then screened on 0.8% agar plates containing diluted (50%, v/v) Basta 50 mg/L. Transgenic T3 lines were isolated and selected based on their segregation ratios for Basta resistance.

### 2.7. Growth and Phenotypic Evaluation of Transgenic Plants

For *A. thaliana* transformation and phenotypic analysis of adult transgenic plants, seeds were sown directly in soil and incubated in an artificial climatic chamber at 22 °C with a 16/8 h (day/night) photoperiod. PCR analysis using gene-specific primers (Table S1) was employed to confirm the presence of the transgenes in the transformed plants. For phenotypic analysis of the seedlings, seeds of *A. thaliana* wild type (WT) and transgenic T3 lines (homozygous with single copy of transgene) were surface-sterilized and grown on half Murashige & Skoog Medium (MS) solid plates. Seeded plates were kept at 4 °C for two days, and were then vertically incubated at 22 °C with a 16/8 h (day/night) photoperiod for the indicated periods. At least ten individual seven-day-old seedlings of each transgenic line were applied for root hair phenotype survey (root hair number per 2 nm near the root elongation zone) using the OLYMPUS MVX10 (Olympus, Tokyo, Japan). The assays were repeated thrice (*n* = 3, 10 plants/genotype/experiment) with similar results. One-way Analysis of Variance (ANOVA) test was performed to assess the statistical differences among the mean values (least significant difference test; *p* < 0.05) using SPSS v.20.

## 3. Results and Discussion

### 3.1. Identification and Phylogenetic Analysis of BnMYB Proteins

A total of 680 non-redundant putative *BnMYB*s were identified in *B. napus* Darmor–*bzh* [24] including 227 *BnMYBR* (33.4%), 429 *Bn2R-MYB* (63.1%), 22 *Bn3R-MYB* (3.2% including 17 typical *Bn3R-MYB* and five *Bn3R-MYB-like* genes) and two *Bn4R-MYB* (0.3%) genes. These *B. napus* candidates represent ~3.4-found of the total number of *AtMYB*s in *A. thaliana* [22]. Moreover, to ensure the integrity of our data, we further searched in the *B. napus* ZS11 genome [29], and compared the sequence similarities and identities of the candidates identified in the *B. napus* Darmor–*bzh* and ZS11 cultivars. Results revealed that most of the MYB proteins of the two ecotypes had >90% similarity levels, and the sequence annotation information from Darmor–*bzh* was more complete (Table S2). Therefore, the candidates of each *MYB*-type family annotated from Darmor–*bzh* were used in the present study for further analyses, which were named according to their genome locus (Table S2). The physicochemical property of the 680 candidates is shown in Table S2.

**Figure 1:**
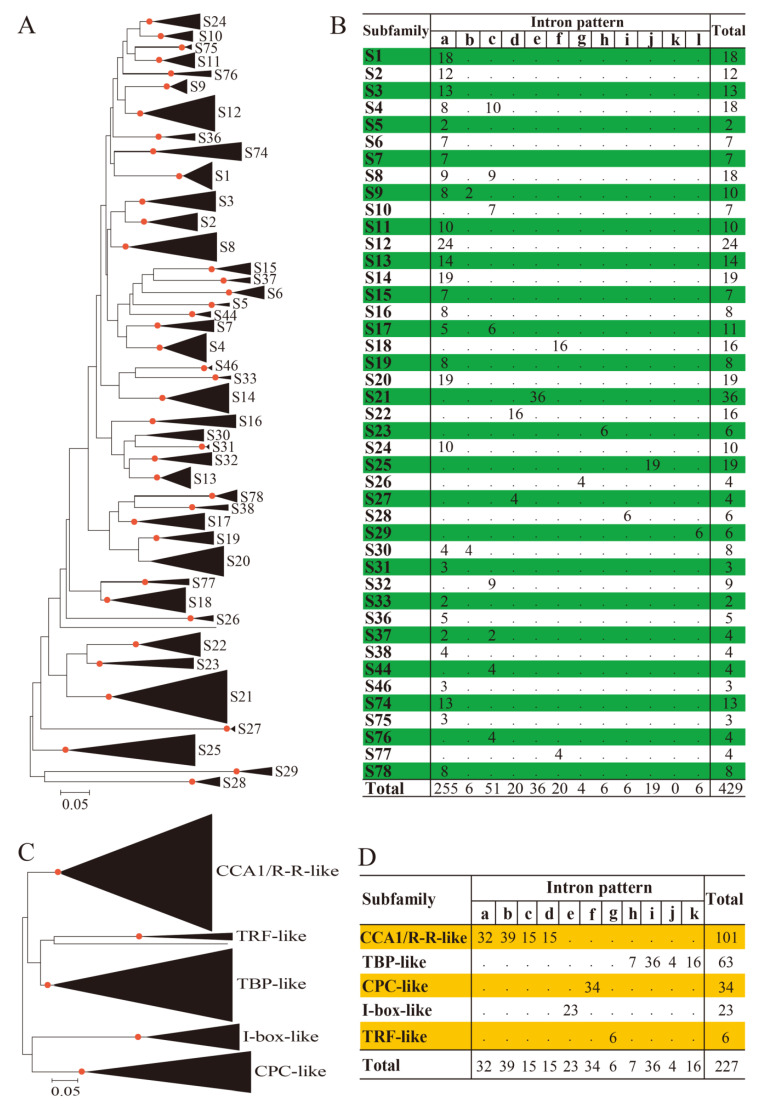
Phylogenetic relationships and intron patterns of MYBR and 2R-MYB proteins from *Brassica napus* (Bn) and *Arabidopsis thaliana* (At). The unrooted phylogenetic trees were constructed using the neighbor-joining (NJ) method based on the alignment of the MYB domains of the 429 *B. napus* 2R-MYBs and 127 *A. thaliana* 2R-MYBs (including the CDC5 like proteins), and the MYB-like domains of the 227 *B. napus* and 68 *A. thaliana* MYBR proteins, respectively. Nodes with bootstrap values ≥70% are dotted in red. The scale shows the relative differences in the examined sequences. Subfamilies are represented in compressed subtree by black triangles with both depth and width proportional to sequence divergence and size, respectively. (**A**) The NJ tree of 556 2R-MYB proteins from *B. napus* (429 proteins) and *A. thaliana* (127 proteins), which were divided into 43 subfamilies (S1–S33, S36–S38, S44, S46, and S74-S78). (**B**) Summary of intron patterns identified for the 43 *B. napus 2R-MYB* subfamilies. Intron patterns ‘d–h’ and ‘l’ were designated by referring to Du et al. 2015 [5]. (**C**) The NJ tree of 295 MYBR proteins from *B. napus* (227 proteins) and *A. thaliana* (68 proteins), which were clustered into five subfamilies, namely CCA1/R-R-like, TBP-like, CPC-like, I-box-like and TRF-like. (**D**) Summary of intron patterns identified for the five *B. napus MYBR* subfamilies. Intron pattern designation (‘a–k’) was referred to Du et al. 2013 [4].

Given the limited number (generally one to three members in many plant species) and high sequence homology of the 3R-MYB and 4R-MYB proteins, in this study, we focused on the evolutionary relationships of the BnMYBR and Bn2R-MYB families by constructing separate unrooted NJ trees for the members of these two families, based on the multiple alignments of their MYB domains. We also included AtMYBRs and At2R-MYBs from *A. thaliana* into the respective phylogenetic analysis (Figure S1). For subfamily classification, we considered the results received from *A. thaliana* [22] and our previous studies in 50 eukaryotes [4,5]. With respect to the Bn2R-MYBs, based on the bootstrap values and topology of the phylogenetic tree, the candidates were classified into 43 subfamilies (Figure 1A and Figure S1), 38 of which were consistent with our previous studies [5], strengthening that the 2R-MYBs are distributed in a conserved way in many plant species. Some of the Bn2R-MYBs and previously reported At2R-MYBs encoded by *‘*orphan’ genes were clustered into five new subfamilies (S74–S78) with strong bootstrap support (=100%) (Figure 1A and Figure S1), which indicates the presence of some previously neglected Brassicaceae-specific subfamilies. The numbers of Bn2R-MYBs in each subfamily were different, ranging from two to 36 members (Figure 1B and Figure S1). For example, there are 36 and 24 members in subfamilies S21 and S12, respectively, whereas there are only two members in S5 and S33 (Figure 1B and Table S2). This result shows that there was a clear expansion bias of *Bn2R-MYB*s in different subfamilies, implying that the gene retention and loss rates of different *Bn2R-MYB* subfamilies after duplications were diverse during the evolution.

Next, we analyzed the intron patterns including the intron distribution, positions, and phases over the genomic regions encoding the MYB domains of the Bn2R-MYBs, which revealed conserved intron patterns. Furthermore, according to the data on the absolute conserved intron insertion positions and phases, the gene structures of the MYB domains of Bn2R-MYBs were divided into 11 conserved intron patterns (‘a–l’, while pattern ‘k’ is lacking) that were highly conserved in each subfamily (Figure 1B and Table S2). This result was consistent with the result of our previous study of *2R-MYB*s identified in 50 major eukaryotic species [5]. Thus, in this study, we followed the intron pattern designation previously reported [5]. As a result, 34 of the 43 subfamilies contained patterns ‘a–c’ accounting for ~73% of *Bn2R-MYB*s (a = 59.4%, b = 1.4%, c = 11.9%), whereas the remaining nine subfamilies contained patterns ‘d–h’ and ‘l’ (~27% of *Bn2R-MYB*s). Four of the five newly identified subfamilies (S74–S76 and S78) had patterns ‘a’ or ‘c’, whereas S77 shared pattern ‘f’ with S18 (Figure 1B and Table S2). These results demonstrated that the intron patterns of *Bn2R-MYB*s were highly conserved in each subfamily. The gene numbers and expansion trends of different subfamilies were quite diverse, where the rapid expansion and preferential retention of this gene family mainly occurred in genes with patterns ‘a–c’, especially pattern ‘a’.

The BnMYBRs were classified into five major subfamilies: CCA1/R-R-like, I-box-like, CPC-like, TRF-like, and TBP-like (Figure 1C and Figure S2). Unlike the *Bn2R-MYB*s, no species-specific subfamilies and/or clades were observed, implying that the *BnMYBR* family was more conserved than the *Bn2R-MYB* family during evolution. CCA1-like/R-R (101 members) and TBP-like (63 members) were the largest subfamilies, and could be further divided into several clades with different intron patterns (Figure S2 and Table S2). All members of the CCA1-like/R-R subfamily contained the common motif SHAQK(Y/F)F. Similar to the *Bn2R-MYB*s, intron patterns in the regions spanning MYB domains encoded by the *BnMYBR*s of this subfamily differed, but were highly conserved in each of the four major clades (patterns ‘a–d’) (Figure 1D). Similarly, the reliability of the TBP-like subfamily was well supported by the presence of the consensus motif LKDKW(R/K)(N/T), and the intron patterns ‘h–k’ were conserved in each clade (Table S2). The CPC-like subfamily (34 members) was characterized by only having a MYB domain without a transcriptional activation domain, and all members shared intron pattern ‘f’. The I-box-like subfamily consisted of 23 members sharing intron pattern ‘e’, whereas the TRF-like subfamily was the smallest (six members) with intron pattern ‘g’. It is worth mentioning that no *BnMYBR* homologs of the ’orphan‘ genes *AtMYBR23* and *AtMYBR48* were identified in *B. napus*. The diverse intron patterns in CCA1-like/R-R and TBP-like *BnMYBR* subfamilies were consistent with the large numbers of their members and multiple separated clades, which indicates the expansion and functional diversification trend in the *BnMYBR* family. In contrast, members in the last three *BnMYBR* subfamilies were relatively conserved during evolution in terms of conserved gene number and gene structure (intron patterns).

### 3.2. Diversification of 2R-MYB Genes in Higher Plants

As shown in Figure 1A, some of the 429 *Bn2R-MYB*s were clustered into five new subfamilies (S74–S78) alongside several *A. thaliana ‘*orphan’ genes (Figure S1), indicating the expansion trend of the *Bn2R-MYB* family, and perhaps functional diversification of its members. Genome-wide analyses of the *2R-MYB* gene family have been carried out in many plant species such as *A. thaliana* [22], rice [23], *Populus trichocarpa* [40], *Ananas comosus* [41], and potato (*Solanum tuberosum*) [42]. The classification of this gene family in these studies commonly refers to the results obtained from *A. thaliana* [22], which includes 25 main subfamilies. Based on the analysis of 50 major eukaryotic lineages, we found many species- or lineage-specific subfamilies in land plants [5]. These results imply that the distribution and classification of this gene family are much wider than we have ever expected.

**Figure 2:**
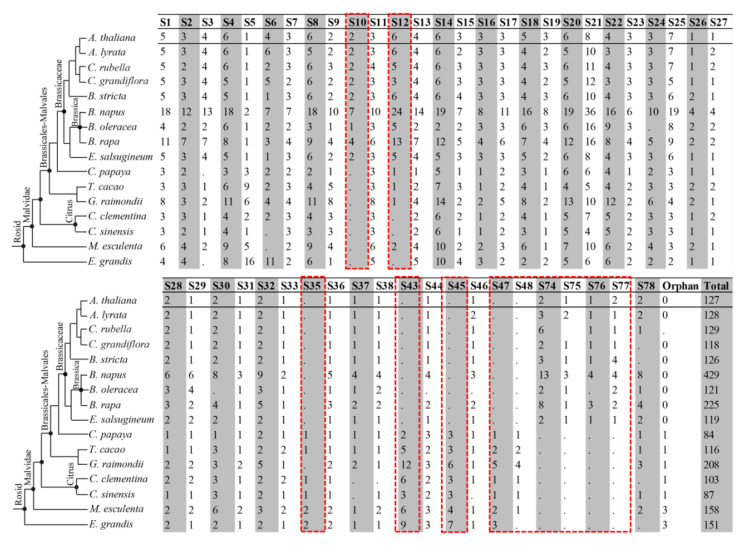
Presence/absence of the *2R-MYB* genes in 16 representative plant genomes by phylogenetic position. Numbers of *2R-MYB* genes in each subfamily (S1–S78) are shown. Red boxes indicate the lineage-specific subfamilies present or absent in nine Brassicaceae species.

To gain further insights into the evolutionary mechanisms of the *2R-MYB*s in Brassicaceae, we identified putative *2R-MYB* genes in eight other Brassicaceae and seven out-lineages in Phytozome including *E. grandis* v2.0, *M. esculenta* v6.1, *C. clementina* v1.0, *C. papaya ASGPB* v0.4, *G. raimondii* v2.1, *T. cacao* v1.1, *A. lyrata* v1.0, *A. thaliana* TAIR10, *C. grandiflora* v1.1, *C. rubella* v1.0, *E. salsugineum* v1.0, *B. stricta* v1.2, *B. rapa FPsc* v1.3, and *B. oleracea* for a phylogenetic analysis. The non-redundant set of *2R-MYB*s from *C. sinensis* (87 genes) was taken from our previous report [5]. Finally, a total of 2429 typical *2R-MYB*s were identified in these 16 species including *B. napus* (Figure 2 and Table S3), and a NJ tree was constructed based on the multiple alignment of their MYB domains (Figure S3). The topology of this NJ tree was mostly the same as that obtained in our previous study for 50 selected species [5]. Therefore, to avoid confusion in terms of subfamily names, in this study, we retained the same nomenclature, which was used for *A. thaliana* [22] and in our previous study [5], for classification of the *2R-MYB* members.

Phylogenetic analysis revealed that many *2R-MYB*s representing various classes showed very high homology within or across species that were grouped as sister pairs (Figure S3). Generally, the 2429 putative *2R-MYB*s from different species were clustered in many compact clades with high support values. Most subfamilies contained members from all of the 16 species investigated, and 38 of the 73 subfamilies defined in eukaryotes in our previous study [5] were well supported in the new tree, demonstrating the conservation of these subfamilies during the evolution. Meanwhile, some newly identified *2R-MYB*s and the previously defined *A. thaliana* ’orphan‘ genes (*AtMYB039*, *AtMYB047,* and *AtMYB049*) were clustered into seven new subfamilies that had been previously neglected. Furthermore, two subfamilies containing candidates from Malvidae, except *Brassica*, were classified as S77 and S78 (Figure 2). In addition, homologs of *AtMYB048* and *AtMYB104*, previously classified into S38 and S18, were reclassified into S77 and S78, respectively, based on topology and bootstrap values. Four of the newly identified subfamilies (S74, S75, S76, and S77) and S10 were likely to be *Brassica*-specific because no counterparts were identified in other species (Figure 2 and Figure S3). Similarly, the previously defined *A. thaliana*-specific subfamily S12 [22,42,43], which is specifically involved in the glucosinolate biosynthesis pathway [42], was demonstrated to be distributed only in the nine Brassicaceae species and *M. esculenta* among the 16 plant species investigated in this study. Conversely, five subfamilies (S35, S43, S45, S47, and S48) were absent from the nine investigated Brassicaceae species. Our results suggested that there are many more lineage-specific subfamilies of *2R-MYB*s in plants that exhibited lineage-specific expansion or deficiency (Figure 2 and Figure S3).

Recently, based on the analyses in 35 plant species, 18 rosid-specific subfamilies/clusters/clades (e.g., S1, S3–5, S12, etc.), four asterid-specific subfamilies (S39, S40, S43, and S80), and nine subfamilies (S29–36 and S69) specific to one of the 35 investigated species were identified [43]. Similarly, many subfamilies/clusters/clades specific to different lineages and species were found in numerous studies [42,43,44,45,46,47]. For example, subfamily S29 was proven to harbor only potato genes [42], seven subfamilies (e.g., S1, S5, S11, etc.) contained only *2R-MYB* genes of *Sesamum indicum* [44], S26 harbored only *2R-MYB* genes from *Ginkgo biloba* [45], 12 subfamilies (e.g., S5, S25, S32, etc.) contained only grass-species *2R-MYB* genes, and S15 and S18 subfamilies possessed only *Phyllostachys edulis 2R-MYB* genes [46]. These results suggest that lineage- or species-specific *2R-MYB*s may be genomic relics that have evolved independently or may have unique function(s) in particular lineage or species, implying that expansion and diversification of *2R-MYB*s occurred during the evolution. Although the functions of these subfamilies remain unknown, our analysis provided evidence to support that some members of the *2R*-*MYB* gene family might have evolved to fulfill lineage-specific functions [47,48].

### 3.3. Chromosomal Distribution and Expansion of the MYB Members in B. napus

To explore the duplication events of each type of the *B. napus MYB* superfamily, we analyzed their chromosomal distribution and colinear relationships with their respective counterparts in *B. rapa*, *B. oleracea,* and *A. thaliana.* Chromosomal distribution analysis showed that 429 *Bn2R-MYB*s were distributed on all 19 *B. napus* chromosomes (Figure S4A). There were 213 and 216 *Bn2R-MYB*s on A_n_ and C_n_ subgenomes, respectively. In subgenome A_n_, 16, 21, 37, 9, 14, 26, 22, 14, 21, and 12 *Bn2R-MYB*s were located on chromosomes A01–A10, respectively, while the chromosomal locations of the remaining 21 genes remain to be determined. In subgenome C_n_, 14, 21, 31, 15, 16, 15, 23, 19, and 25 *Bn2R-MYB*s were distributed on chromosomes C01–C09, respectively, while the chromosomal locations of the remaining 37 genes remain unclear. High densities of the *Bn2R-MYB*s were observed on the top and/or bottom end(s) of each chromosome such as C03 and C07–C09 (Figure S4A). Thus, the distributions of *Bn2R-MYB*s across the 19 chromosomes were uneven. A similar trend was observed in the *BnMYBR* family. The 227 *BnMYBR*s were also distributed on all 19 chromosomes, with 114 and 113 on subgenomes A_n_ and C_n_, respectively (Figure S4B). In both the A_n_ and C_n_ subgenomes, chromosome 3 contained the largest number of candidates (19 and 22 genes, respectively). Furthermore, the numbers of *BnMYBR*s were also higher on the top and/or bottom end(s) of each chromosome. As for the 22 *Bn3R-MYB*/*Bn3R-MYB-like* genes, 11 and 11 genes were located on the A_n_ and C_n_ subgenomes, respectively (Table S2). With respect to the two *Bn4R-MYB*s, one is located on chromosome A05 (*Bn4R-MYB1*) and another on chromosome C01 (*Bn4R-MYB2*) (Table S2).

Collinearity analysis showed that the *B. napus* A_n_ and C_n_ subgenomes were widely colinear to the corresponding diploid *B. rapa* and *B. oleracea* genomes, respectively. Accordingly, many orthologous gene pairs between the A_n_ and C_n_ subgenomes and their respective progenitor genomes were observed (Table S4). A total of 362 of the 429 (~84%) *Bn2R-MYB*s were involved in colinear relationships in *B. napus* (Figure S4A and Table S4). Among the 362 *Bn2R-MYB*s, 126 (35%) had colinear relationships with *2R-MYB*s of *B. rapa* (96 genes, ~76%) and/or *B. oleracea* (30 genes, ~24%). Thus, many *Bn2R-MYB*s were derived from the two progenitor genomes, with more genes from *B. rapa*. After the allopolyploidy event, 68 (~19%), 109 (~30%), and 59 (~16%) of the 362 *Bn2R-MYB*s underwent SE, HE, and SD events, respectively, while no TD events were identified. These results demonstrated that the small-scale duplication events (including SE, HE, and SD) might also play a major role for the large gene expansion of the *Bn2R-MYB* family in the *B. napus* genome.

Of the 227 *BnMYBR*s, 182 (~80%) genes exhibited colinear relationships in *B. napus*. Out of these 182 *BnMYBR*s, 107 (~59%) and 55 (~30%) genes were involved in colinear relationships with *MYBR* genes of *B. rapa* and *B. oleracea*, respectively (Figure S4B and Table S4). This result indicates that the majority of *BnMYBR*s were obtained through the allopolyploidy event, and the *MYBR* genes derived from *B. rapa* tended to be retained in *B. napus.* Furthermore, 32 (~18%), 32 (~18%), and 20 (~11%) of the 182 *BnMYBR*s (~18%) underwent SE, HE, and SD events, respectively, while no TD events were identified. Of the 22 *Bn3R-MYB*/*Bn3R-MYB-like* genes, 19 (~86%) exhibited colinear relationships in *B. napus*, with 11 and five genes also showing colinear relationships with *3R-MYB* genes in *B. rapa* (50%) and *B. oleracea* (23%), respectively. This finding indicates that the majority of *Bn3R-MYB*s were also obtained from the allopolyploidy event. Additionally, one (5%), eight (36%), and three (14%) of the 19 *3R-MYB* genes were derived from the SE, HE, and SD events, respectively, while no *3R-MYB* genes were involved in any TD event (Table S4).

Overall, our results collectively demonstrated that large-scale duplication event (allopolyploidy) and small-scale duplication events (SD, SE, and HE) were the major forces driving the large expansion of the *MYB* superfamily in *B. napus*. After allopolyploidy, many SE and HE events occurred across the A_n_ and C_n_ subgenomes, resulting in a similar number of genes for all *MYB*-type genes in both subgenomes of *B. napus* with higher preference for the retention of *MYB* genes from *B. rapa*. On the contrary, TD events likely had less effect on the expansion of *BnMYB*s in *B. napus*.

### 3.4. Conserved Functions of B. napus WER Homologs in Root Hair Development in A. thaliana

To date, many *MYBs*, especially *2R-MYB*s, have been experimentally demonstrated to play key roles in root development. For example, *AtMYB30* and *AtMYB60* of S1 have been shown to regulate root elongation [49,50], *AtMYB36* of the S14 subfamily is involved in lateral root primordium development [51], *AtMYB066/WER* homologs of S15 are well-known in regulating root hair patterning [10,52], *AtMYB33*, *AtMYB65*, and *AtMYB101* of S18 have been reported to regulate primary root growth [53], *AtMYB77* of S22 interacted with auxin response factors (ARFs) to regulate lateral root formation [54], *AtMYB93* of S24 acted as a negative regulator of lateral root development [55], *AtMYB88* of S28 regulated root gravitropism [56], and *AtMYB59* of S38 could inhibit root growth by extending the metaphase of mitotic cells [57].

Root hair patterning in *A. thaliana* is controlled by the interplay of several TFs including the *2R-MYB*-type *WER* gene [10]. Our phylogenetic analysis showed that seven *Bn2R-MYB*s (*BnMYB019*, *BnMYB042*, *BnMYB077*, *BnMYB118*, *BnMYB189*, *BnMYB231,* and *BnMYB388*) and three *A. thaliana WER/AtMYB066* homologs (*AtMYB066*, *AtMYB023*, and *AtMYB000*) were clustered in the S15 subfamily (Figure S1). Among them, *BnMYB019*, *BnMYB189*, *BnMYB231,* and *BnMYB388* are the homologs of *WER*; thus, they were named *BnWER*s. To further explore the roles of these four *BnWER*s in root hair development, we introduced them into *A. thaliana* WT and/or *wer* mutant plants under the control of the *35S* promoter. At the same time, *BnMYB019* and *BnMYB231* genes directed by their native promoter were also used to transform *A. thaliana wer* mutant plants.

**Figure 3:**
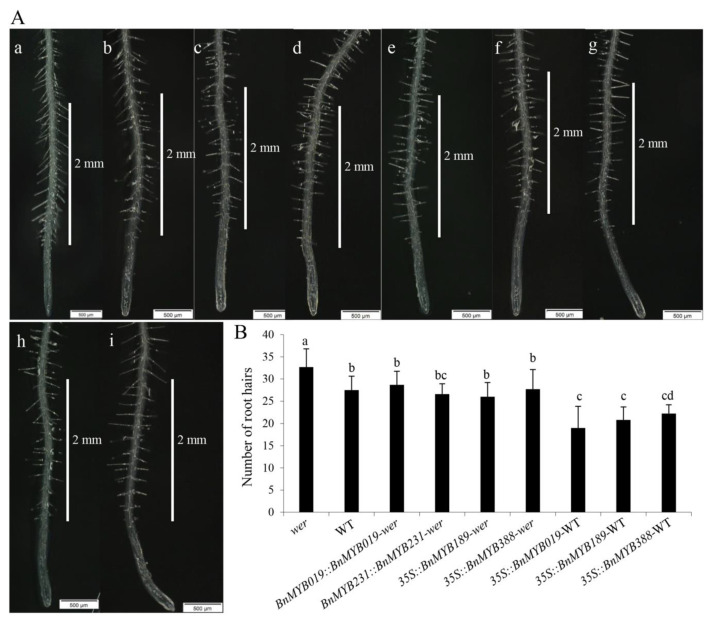
Complementation assay of root hair density in the *Arabidopsis thaliana wer* mutant background using the four *Brassica napus WER*-homologous genes *BnMYB019*, *BnMYB189*, *BnMYB231,* and *BnMYB388*. (**A**) Representative picture showing root hair morphology and density (2 nm) in 7-day-old *A. thaliana* seedlings of *wer* mutant (**a**), wild-type (WT) (**b**), and *wer* mutant plants harboring *BnMYB019::BnMYB019* (**c**), *wer* mutant plants harboring *BnMYB0231::BnMYB231* (**d**), *wer* mutant plants harboring *35S::BnMYB189* (**e**), *wer* mutant plants harboring *35S::BnMYB388* (**f**), WT plants harboring *35S::BnMYB019* (**g**), WT plants harboring *35S::BnMYB189*, and (**h**) WT plants harboring *35S::BnMYB388* (**i**). (**B**) Root hair density in 7-day-old *A. thaliana* seedlings of the corresponding lines (*n* = 3, 10 plants/genotype/experiment). Different letters indicate significant differences (least significant difference test; *p* < 0.05).

The *wer* mutant had more root hairs than WT as reported earlier [10] (Figure 3A,B). The T3 homozygous lines ectopically expressing *BnMYB189* or *BnMYB388* in *wer* background using the *35S* promoter showed reduced root hair numbers that were comparable to that of WT plants (Figure 3A,B). Likewise, all homozygous transgenic lines harboring the *BnMYB019::BnMYB019* or *BnMYB231::BnMYB231* construct in the *wer* background completely rescued the aberrant root hair density of the *wer* mutant (Figure 3A,B). These results indicated that all four of the examined *BnMYB019*, *BnMYB189*, *BnMYB231,* and *BnMYB388* genes have conserved functions with the *A. thaliana WER* gene in regulating root hair formation. Furthermore, the transgenic lines carrying the *35S::BnMYB019*, *35S::BnMYB189* or *35S::BnMYB388* construct in the WT background displayed lower root hair numbers than the WT plants (Figure 3A,B), strengthening their negative regulatory roles in root hair development.

Taken together, our results demonstrated that the *Bn2R-MYB* homologs in the S15 subfamily shared a conserved function in root hair development, and could complement the loss-of-function of the *A. thaliana WER* gene. This finding supports that in most cases, genes in a same subfamily with high similarity in sequence and expression patterns tend to have functional redundancy, indicating a positive correlation among expression patterns, sequence features, and gene functions across different species such as *B. napus* and *A. thaliana*.

### 3.5. Hormone-Induced Expression Profiling of B. napus MYB Genes by RNA-Seq

**Figure 4:**
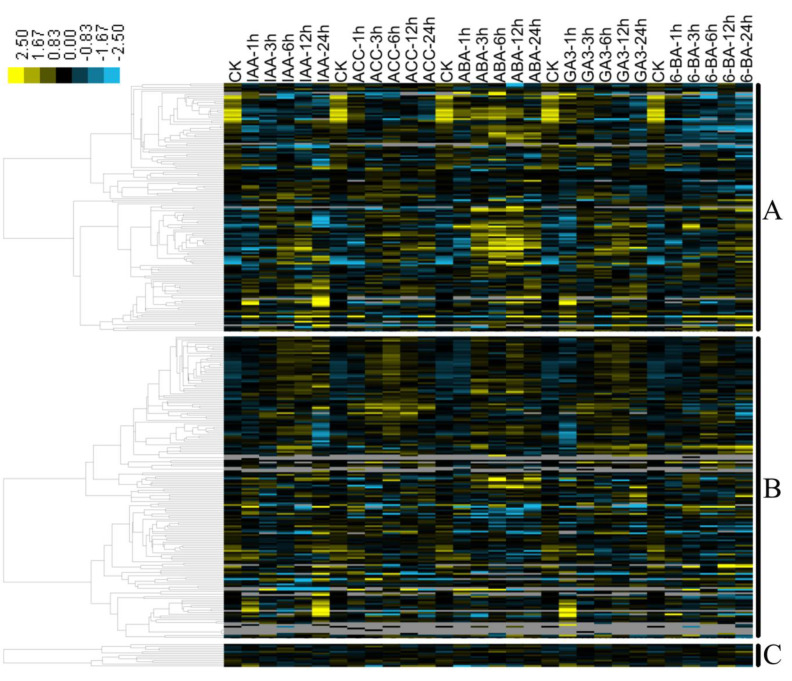
Transcript profiles of members of the *B. napus MYB* superfamily in seedling roots under individual treatments with auxin (IAA), gibberellin (GA_3_), cytokinin (6-BA), abscisic acid (ABA) and ethylene (ACC) in a time-course manner. (A–C) Expression patterns of 141 *Bn2R-MYB* (**A**), 171 *BnMYBR* (**B**) and 12 *Bn3R-MYB* (**C**) genes in roots of *B. napus* seedlings under the IAA, ACC, ABA, GA_3_ and 6-BA treatments. Transcript data were obtained by RNA-Seq. Log_2_ (FPKM ≥ 1) values were displayed according to the color bar (top left). *BnMYB*s with no or weak expression (FPKM < 1) in all samples were excluded from this figure. Expression values of all *BnMYB*s under hormone treatments are supplied in Table S5. CK, control (non-treatment at 0 h).

It was demonstrated that the functions of *MYB* genes in plant development, stress responses, and metabolism were generally mediated by phytohormones. For example, homologs of S18 (e.g., *A. thaliana AtMYB33* and *AtMYB65*, *G. hirsutum GhMYB24*, and *H. vulgare HvGAMYB*) were shown to regulate anther/pollen development by the GA signal [58,59,60]. *AtMYB21*, *AtMYB24,* and *AtMYB57* of S19 play roles in stamen development in a jasmonate-dependent manner [61,62], while *AtMYB77* of S22 regulates lateral root growth through modulating auxin signal transduction [54]. In the present study, we performed a global expression profiling of the *B. napus MYB* superfamily under ABA, IAA, GA_3_, 6-BA, and ACC treatments in a time-course manner to identify those *BnMYB*s whose functions in *B. napus* might be mediated by hormones (Figure 4).

Regarding the *Bn2R-MYB* family (429 members), 141 genes (~33%) showed detectable transcript levels (FPKM ≥ 1) in the roots under hormone treatments, whereas the remaining genes had weak (FPKM < 1) or no transcript accumulation (Figure 4A and Table S5). The expression profiles of these 141 *Bn2R-MYB*s, which are distributed into 25 of the 43 *Bn2R-MYB* subfamilies, were classified into three major patterns. The first group (73 genes) was evidently upregulated (≥1.5-fold) by all five hormones and under most of the treatment conditions, especially ABA (e.g., *Bn2R-MYB126* and *Bn2R-MYB170* in S4, *Bn2R-MYB189* and *Bn2R-MYB252* in S9, *Bn2R-MYB148*, *Bn2R-MYB341* in S11, etc.). The second group (51 genes) was evidently downregulated (≥1.5-fold) by all five hormones and in most of the treatment conditions, especially the S22 members (e.g., *Bn2R-MYB010*, *Bn2R-MYB127,* and *Bn2R-MYB330*). The members of the third group (20 genes) had their expression altered by a few hormones, for example, *Bn2R-MYB209* in S78, *Bn2R-MYB331* in S28, and *Bn2R-MYB322* in S1 were upregulated (≥1.5 fold change) by IAA, ABA and/or GA_3_ treatments, while *Bn2R-MYB334* in S1, *Bn2R-MYB299* in S12, and *Bn2R-MYB001* and *Bn2R-MYB215* in S22 were downregulated by GA_3_ and/or 6-BA treatments (Figure 4A and Table S5). The different expression patterns of the *Bn2R-MYB*s suggest their diverse roles in responses to hormones. Accordingly, consistent with the diverse hormone-induced expression profile, homologs of S1 were demonstrated to be involved in ABA-mediated defense (e.g., against drought) and development (e.g., roots) [14,50,63], members of S22 that regulate plant responses to abiotic stresses (e.g., drought, salt, and cold stresses) [13] and lateral root development [64] were also ABA-mediated, while *AtMYB88* in S28, which was involved in stomatal formation and root gravitropism, was auxin-mediated [56]. Although only a few members of the *MYB* superfamily have been functionally characterized in plants to date, our results suggest that their potential roles in root-related processes may be commonly hormone-mediated.

With respect to the *BnMYBR* family, 171 of the 227 genes (75%) exhibited detectable transcript levels (FPKM ≥ 1) in roots under hormone treatments (Figure 4B and Table S5). This ratio was much higher than that of the *Bn2R-MYB* family, suggesting that *BnMYBR*s may be more sensitive to exogenous hormone treatments. Among the five subfamilies, members of the CCA1/R-R-like, TBP-like, and TRF-like subfamilies tended to be expressed in roots under various hormone treatments. Except for a few genes (e.g., *BnMYBR099*, *BnMYBR128*, and *BnMYBR077*), the majority of *BnMYBR*s (130 genes) were upregulated (≥1.5-fold) at several time points by at least one of the five hormones, and similar expression patterns were often observed for homologs in the same subfamily or clade (Figure 4B and Table S5). On the other hand, 41 *BnMYBR*s were downregulated (≥1.5-fold) by at least one of the five hormones (Figure 4B and Table S5), many of which, mainly the CCA1/R-R-like subfamily genes such as *BnMYBR103*, *BnMYBR132*, and *BnMYB179* were downregulated by all five hormones under all treatment conditions.

As for the 22 *Bn3R-MYB* and *Bn3R-MYB-like* genes, 12 of the 17 *Bn3R-MYB*s (71%), and one of the five *Bn3R-MYB-like* genes (20%) exhibited detectable transcript levels (FPKM ≥ 1) in roots upon hormone treatments (Figure 4C and Table S5). Out of these 13 *Bn3R-MYB* and *Bn3R-MYB-like* genes, six and seven genes were found to be upregulated and/or downregulated, respectively, by hormone treatments (Figure 4C and Table S5). For example, *Bn3R-MYB08* was upregulated by both ACC and ABA treatments, *Bn3R-MYB07* was induced by IAA, ABA, and 6-BA treatments, while *Bn3R-MYB10* was upregulated by IAA, ABA, and 6-BA treatments (Table S5). None of the two identified *Bn4R-MYB*s had detectable expression levels in any hormone-treated samples (Table S5). These data together suggest that the *3R-MYB*, *Bn3R-MYB-like*, and *Bn4R-MYB* genes may be less affected by hormones.

## 4. Conclusions

In this study, we systematically identified and classified the *BnMYB* superfamily. Exon–intron structure analyses of this superfamily confirmed the conserved exon–intron structures within the conserved MYB domain. Collinearity analyses revealed that allopolyploidy between *B. rapa* and *B. oleracea* mainly contributed to the large *BnMYB* gene expansion in *B. napus*. Comparative analysis of the *2R-MYB* families in 16 Brassicaceae and non-Brassicaceae species identified five Brassicaceae-specific subfamilies (S10, S74-77) and five subfamilies (S35, S43, S45, S47, and S48) that are absent from the nine examined Brassicaceae species. In addition, one subfamily (S12) was found to be merely distributed in the nine Brassicaceae species and *M. esculenta*. Functional analysis of the four *WER*-homologous *Bn2R-MYB*s in a complementary assay indicated their conserved roles in root hair patterning. Global expression profiling demonstrated that the *Bn2R-MYB*s and *BnMYBR*s were widely responsive to hormone treatments in roots, suggesting that the hormone-responsive *BnMYB*s may regulate biological processes in *B. napus* in a hormone-dependent manner.

## Supplementary Materials

The following are available online at https://www.mdpi.com/2218-273X/10/6/875/s1. Figure S1: Phylogenetic tree of the R2R3-MYB proteins (2R-MYBs) in *Brassica napus* and *Arabidopsis thaliana*; Figure S2: Phylogenetic tree of the MYB-related proteins (MYBRs) from *Brassica napus* and *Arabidopsis thaliana*; Figure S3: Phylogenetic tree of 2429 plant 2R-MYB proteins from 16 plant species; Figure S4: Physical positions and duplications of 429 *Bn2R-MYB* and 227 *BnMYBR* genes in 19 *Brassica napus* chromosomes; Table S1: Primers used in this study; Table S2: The *MYB* genes identified the *Brassica napus* genome; Table S3: The *2R-MYB* genes identified in 15 plant species (excepting *Brassica napus*) used in this study; Table S4: Duplications of the *MYB* genes in the *Brassica napus*, *B. rapa*, and *B. oleracea* genomes; Table S5: Expression value (FPKM) of the *Brassica napus MYB* genes under five hormone treatments in roots.

## References

[1] Martin C., Paz-Ares J. (1997). MYB transcription factors in plants. Trends Genet..

[2] Lipsick J.S. (1996). One billion years of Myb. Oncogene.

[3] Dubos C., Stracke R., Grotewold E., Weisshaar B., Martin C., Lepiniec L. (2010). MYB transcription factors in *Arabidopsis*. Trends Plant Sci..

[4] Du H., Wang Y.B., Xie Y., Liang Z., Jiang S.J., Zhang S.S., Huang Y.B., Tang Y.X. (2013). Genome-wide identification and evolutionary and expression analyses of MYB-related genes in land plants. DNA Res..

[5] Du H., Liang Z., Zhao S., Nan M.G., Tran L.S., Lu K., Huang Y.B., Li J.N. (2015). The Evolutionary History of R2R3-MYB Proteins across 50 Eukaryotes: New Insights into Subfamily Classification and Expansion. Sci. Rep..

[6] Du H., Yang S.S., Liang Z., Feng B.R., Liu L., Huang Y.B., Tang Y.X. (2012). Genome-wide analysis of the MYB transcription factor superfamily in soybean. BMC Plant Biol..

[7] Li S., Wang W., Gao J., Yin K., Wang R., Wang C., Petersen M., Mundy J., Qiu J.L. (2016). MYB75 Phosphorylation by MPK4 Is Required for Light-Induced Anthocyanin Accumulation in *Arabidopsis*. Plant Cell.

[8] Stracke R., Ishihara H., Huep G., Barsch A., Mehrtens F., Niehaus K., Weisshaar B. (2007). Differential regulation of closely related R2R3-MYB transcription factors controls flavonol accumulation in different parts of the *Arabidopsis thaliana* seedling. Plant J..

[9] Albert N.W., Lewis D.H., Zhang H., Schwinn K.E., Jameson P.E., Davies K.M. (2011). Members of an R2R3-MYB transcription factor family in Petunia are developmentally and environmentally regulated to control complex floral and vegetative pigmentation patterning. Plant J..

[10] Lee M.M., Schiefelbein J. (1999). WEREWOLF, a MYB-Related Protein in *Arabidopsis*, Is a Position-Dependent Regulator of Epidermal Cell Patterning. Cell.

[11] Kim J.H., Nguyen N.H., Jeong C.Y., Nguyen N.T., Hong S.W., Lee H. (2013). Loss of the R2R3 MYB, AtMyb73, causes hyper-induction of the SOS1 and SOS3 genes in response to high salinity in *Arabidopsis*. J. Plant Physiol..

[12] Zhang P., Wang R., Ju Q., Li W., Tran L.P., Xu J. (2019). The R2R3-MYB Transcription Factor MYB49 Regulates Cadmium Accumulation. Plant Physiol..

[13] Jung C., Seo J.S., Han S.W., Koo Y.J., Kim C.H., Song S.I., Nahm B.H., Choi Y.D., Cheong J.J. (2008). Overexpression of AtMYB44 enhances stomatal closure to confer abiotic stress tolerance in transgenic *Arabidopsis*. Plant Physiol..

[14] Liao C., Zheng Y., Guo Y. (2017). MYB30 transcription factor regulates oxidative and heat stress responses through ANNEXIN-mediated cytosolic calcium signaling in *Arabidopsis*. New Phytol..

[15] Nagel D.H., Doherty C.J., Pruneda-Paz J.L., Schmitz R.J., Ecker J.R., Kay S.A. (2015). Genome-wide identification of CCA1 targets uncovers an expanded clock network in *Arabidopsis*. Proc. Natl. Acad. Sci. USA.

[16] Nguyen N.H., Lee H. (2016). MYB-related transcription factors function as regulators of the circadian clock and anthocyanin biosynthesis in *Arabidopsis*. Plant Signal. Behav..

[17] Wada T., Tachibana T., Shimura Y., Okada K. (1997). Epidermal cell differentiation in *Arabidopsis* determined by a Myb homolog, CPC. Science.

[18] Pant B.D., Burgos A., Pant P., Cuadros-Inostroza A., Willmitzer L., Scheible W.R. (2015). The transcription factor PHR1 regulates lipid remodeling and triacylglycerol accumulation in *Arabidopsis thaliana* during phosphorus starvation. J. Exp. Bot..

[19] Ito M. (2005). Conservation and diversification of three-repeat Myb transcription factors in plants. J. Plant Res..

[20] Haga N., Kobayashi K., Suzuki T., Maeo K., Kubo M., Ohtani M., Mitsuda N., Demura T., Nakamura K., Jurgens G. (2011). Mutations in MYB3R1 and MYB3R4 cause pleiotropic developmental defects and preferential down-regulation of multiple G2/M-specific genes in *Arabidopsis*. Plant Physiol..

[21] Dai X., Xu Y., Ma Q., Xu W., Wang T., Xue Y., Chong K. (2007). Overexpression of an R1R2R3 MYB gene, OsMYB3R-2, increases tolerance to freezing, drought, and salt stress in transgenic *Arabidopsis*. Plant Physiol..

[22] Stracke R., Werber M., Weisshaar B. (2001). The R2R3-MYB gene family in *Arabidopsis thaliana*. Curr. Opin. Plant Biol..

[23] Katiyar A., Smita S., Lenka S.K., Rajwanshi R., Chinnusamy V., Bansal K.C. (2012). Genome-wide classification and expression analysis of MYB transcription factor families in rice and *Arabidopsis*. BMC Genom..

[24] Zhang C., Ma R., Xu J., Yan J., Guo L., Song J., Feng R., Yu M. (2018). Genome-wide identification and classification of MYB superfamily genes in peach. PLoS ONE.

[25] Qing J., Dawei W., Jun Z., Yulan X., Bingqi S., Fan Z. (2019). Genome-wide characterization and expression analyses of the MYB superfamily genes during developmental stages in Chinese jujube. PeerJ.

[26] Chalhoub B., Denoeud F., Liu S., Parkin I.A., Tang H., Wang X., Chiquet J., Belcram H., Tong C., Samans B. (2014). Plant genetics. Early allopolyploid evolution in the post-Neolithic *Brassica napus* oilseed genome. Science.

[27] Mitchell A., Chang H.Y., Daugherty L., Fraser M., Hunter S., Lopez R., McAnulla C., McMenamin C., Nuka G., Pesseat S. (2015). The InterPro Protein Families Database: The Classification Resource After 15 Years. Nucleic. Acids. Res..

[28] Letunic I., Copley R.R., Schmidt S., Ciccarelli F.D., Doerks T., Schultz J., Ponting C.P., Bork P. (2004). SMART 4.0: Towards genomic data integration. Nucleic. Acids. Res..

[29] Sun F., Fan G., Hu Q., Zhou Y., Guan M., Tong C., Li J., Du D., Qi C., Jiang L. (2017). The high-quality genome of *Brassica napus* cultivar ‘ZS11’ reveals the introgression history in semi-winter morphotype. Plant J..

[30] Tamura K., Peterson D., Peterson N., Stecher G., Nei M., Kumar S. (2011). MEGA5: Molecular evolutionary genetics analysis using maximum likelihood, evolutionary distance, and maximum parsimony methods. Mol. Biol. Evol..

[31] Goodstein D.M., Shu S., Howson R., Neupane R., Hayes R.D., Fazo J., Mitros T., Dirks W., Hellsten U., Putnam N. (2012). Phytozome: A comparative platform for green plant genomics. Nucleic. Acids. Res..

[32] Hu B., Jin J., Guo A.Y., Zhang H., Luo J., Gao G. (2015). GSDS 2.0: An upgraded gene feature visualization server. Bioinformatics.

[33] Bjellqvist B., Hughes G.J., Pasquali C., Paquet N., Ravier F., Sanchez J.C., Frutiger S., Hochstrasser D. (1993). The Focusing Positions of Polypeptides in Immobilized Ph Gradients Can Be Predicted from Their Amino-Acid-Sequences. Electrophoresis.

[34] Chou K.C., Shen H.B. (2008). Cell-PLoc: A package of Web servers for predicting subcellular localization of proteins in various organisms. Nat. Protoc..

[35] Katoh K., Standley D.M. (2013). MAFFT multiple sequence alignment software version 7: Improvements in performance and usability. Mol. Biol. Evol..

[36] Lyons E., Pedersen B., Kane J., Alam M., Ming R., Tang H., Wang X., Bowers J., Paterson A., Lisch D. (2008). Finding and comparing syntenic regions among Arabidopsis and the outgroups papaya, poplar and grape: CoGe with rosids. Plant Physiol..

[37] Cheng F., Liu S., Wu J., Fang L., Sun S., Liu B., Li P., Hua W., Wang X. (2011). BRAD, the genetics and genomics database for Brassica plants. BMC Plant Biol..

[38] De Hoon M.J., Imoto S., Nolan J., Miyano S. (2004). Open source clustering software. Bioinformatics.

[39] Saldanha A.J. (2004). Java Treeview--extensible visualization of microarray data. Bioinformatics.

[40] Wilkins O., Nahal H., Foong J., Provart N.J., Campbell M.M. (2009). Expansion and diversification of the Populus R2R3-MYB family of transcription factors. Plant Physiol..

[41] Liu C., Xie T., Chen C., Luan A., Long J., Li C., Ding Y., He Y. (2017). Genome-wide organization and expression profiling of the R2R3-MYB transcription factor family in pineapple (*Ananas comosus*). BMC Genom..

[42] Li X., Guo C., Ahmad S., Wang Q., Yu J., Liu C., Guo Y. (2019). Systematic Analysis of MYB Family Genes in Potato and Their Multiple Roles in Development and Stress Responses. Biomolecules.

[43] Han Y., Yu J., Zhao T., Cheng T., Wang J., Yang W., Pan H., Zhang Q. (2019). Dissecting the Genome-Wide Evolution and Function of R2R3-MYB Transcription Factor Family in *Rosa chinensis*. Genes.

[44] Mmadi M.A., Dossa K., Wang L., Zhou R., Wang Y., Cisse N., Sy M.O., Zhang X. (2017). Functional Characterization of the Versatile MYB Gene Family Uncovered Their Important Roles in Plant Development and Responses to Drought and Waterlogging in Sesame. Genes.

[45] Liu X., Yu W., Zhang X., Wang G., Cao F., Cheng H. (2017). Identification and expression analysis under abiotic stress of the R2R3-MYB genes in *Ginkgo biloba* L.. Physiol. Mol. Biol. Plants.

[46] Hou D., Cheng Z., Xie L., Li X., Li J., Mu S., Gao J. (2018). The R2R3 MYB Gene Family in *Phyllostachys edulis*: Genome-Wide Analysis and Identification of Stress or Development-Related R2R3MYBs. Front. Plant Sci..

[47] Soler M., Camargo E.L., Carocha V., Cassan-Wang H., Clemente H.S., Savelli B., Hefer C.A., Paiva J.A., Myburg A.A., Grima-Pettenati J. (2015). The *Eucalyptus grandis* R2R3-MYB transcription factor family: Evidence for woody growth-related evolution and function. New Phytol..

[48] Bailey P.C., Dicks J., Wang T.L., Martin C. (2008). IT3F: A web-based tool for functional analysis of transcription factors in plants. Phytochemistry.

[49] Sakaoka S., Mabuchi K., Morikami A., Tsukagoshi H. (2018). MYB30 regulates root cell elongation under abscisic acid signaling. Commun. Integr. Biol..

[50] Oh J.E., Kwon Y., Kim J.H., Noh H., Hong S.W., Lee H. (2011). A dual role for MYB60 in stomatal regulation and root growth of *Arabidopsis thaliana* under drought stress. Plant Mol. Biol..

[51] Liberman L.M., Sparks E.E., Moreno-Risueno M.A., Petricka J.J., Benfey P.N. (2015). MYB36 regulates the transition from proliferation to differentiation in the *Arabidopsis* root. Proc. Natl. Acad. Sci. USA.

[52] Ryu K.H., Kang Y.H., Park Y.H., Hwang I., Schiefelbein J., Lee M.M. (2005). The WEREWOLF MYB protein directly regulates CAPRICE transcription during cell fate specification in the *Arabidopsis* root epidermis. Development.

[53] Xue T., Liu Z., Dai X., Xiang F. (2017). Primary root growth in *Arabidopsis thaliana* is inhibited by the miR159 mediated repression of MYB33, MYB65 and MYB101. Plant Sci..

[54] Shin R., Burch A.Y., Huppert K.A., Tiwari S.B., Murphy A.S., Guilfoyle T.J., Schachtman D.P. (2007). The *Arabidopsis* transcription factor MYB77 modulates auxin signal transduction. Plant Cell.

[55] Gibbs D.J., Voss U., Harding S.A., Fannon J., Moody L.A., Yamada E., Swarup K., Nibau C., Bassel G.W., Choudhary A. (2014). AtMYB93 is a novel negative regulator of lateral root development in *Arabidopsis*. New Phytol..

[56] Wang H.Z., Yang K.Z., Zou J.J., Zhu L.L., Xie Z.D., Morita M.T., Tasaka M., Friml J., Grotewold E., Beeckman T. (2015). Transcriptional regulation of PIN genes by FOUR LIPS and MYB88 during *Arabidopsis* root gravitropism. Nat. Commun..

[57] Mu R.L., Cao Y.R., Liu Y.F., Lei G., Zou H.F., Liao Y., Wang H.W., Zhang W.K., Ma B., Du J.Z. (2009). An R2R3-type transcription factor gene AtMYB59 regulates root growth and cell cycle progression in *Arabidopsis*. Cell Res..

[58] Millar A.A., Gubler F. (2005). The *Arabidopsis* GAMYB-like genes, MYB33 and MYB65, are microRNA-regulated genes that redundantly facilitate anther development. Plant Cell.

[59] Li Y., Jiang J., Du M.L., Li L., Wang X.L., Li X.B. (2013). A cotton gene encoding MYB-like transcription factor is specifically expressed in pollen and is involved in regulation of late anther/pollen development. Plant Cell Physiol..

[60] Murray F., Kalla R., Jacobsen J., Gubler F. (2003). A role for HvGAMYB in another development. Plant J..

[61] Song S., Qi T., Huang H., Ren Q., Wu D., Chang C., Peng W., Liu Y., Peng J., Xie D. (2011). The Jasmonate-ZIM domain proteins interact with the R2R3-MYB transcription factors MYB21 and MYB24 to affect Jasmonate-regulated stamen development in *Arabidopsis*. Plant Cell.

[62] Mandaokar A., Browse J. (2009). MYB108 acts together with MYB24 to regulate jasmonate-mediated stamen maturation in *Arabidopsis*. Plant Physiol..

[63] Lee H.G., Mas P., Seo P.J. (2016). MYB96 shapes the circadian gating of ABA signaling in *Arabidopsis*. Sci. Rep..

[64] Yang Y., Zhang L., Chen P., Liang T., Li X., Liu H. (2020). UV-B photoreceptor UVR8 interacts with MYB73/MYB77 to regulate auxin responses and lateral root development. EMBO J..

