# Supplementary material for: MYB Superfamily in Brassica napus: Evidence for Hormone-Mediated Expression Profiles, Large Expansion, and Functions in Root Hair Development
Source: Biomolecules. 2020 Jun 7;10(6):875. doi: 10.3390/biom10060875 (PMC7356979; doi:10.3390/biom10060875)
Supplement: Supplementary file 1 [file biomolecules-10-00875-s001.zip › Supplementary Materials/Figure S1.pdf]

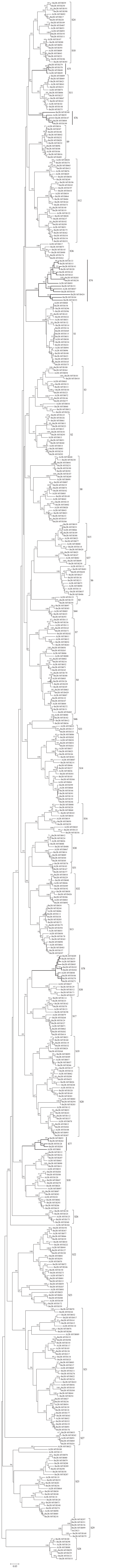

Figure S1. Phylogenetic tree of the R2R3-MYB proteins (2R-MYBs) of *Brassica napus* and *Arabidopsis thaliana*. The unrooted tree was constructed using the neighbor-joining method based on the alignment of the MYB domains of the 429 *B. napus* 2R-MYBs and 127 *A. thaliana* 2R-MYBs (including the CDC5 like proteins). Bootstrap values ≥ 50 (in percentage) are indicated along the branches. The 2R-MYBs are clustered into 43 major subfamilies (S1–S33, S36–S38, S44, S46, and S74-S78). The scale shows relative differences of the examined sequences.
