# Supplementary material for: MYB Superfamily in Brassica napus: Evidence for Hormone-Mediated Expression Profiles, Large Expansion, and Functions in Root Hair Development
Source: Biomolecules. 2020 Jun 7;10(6):875. doi: 10.3390/biom10060875 (PMC7356979; doi:10.3390/biom10060875)
Supplement: Supplementary file 1 [file biomolecules-10-00875-s001.zip › Supplementary Materials/Figure S2.pdf]

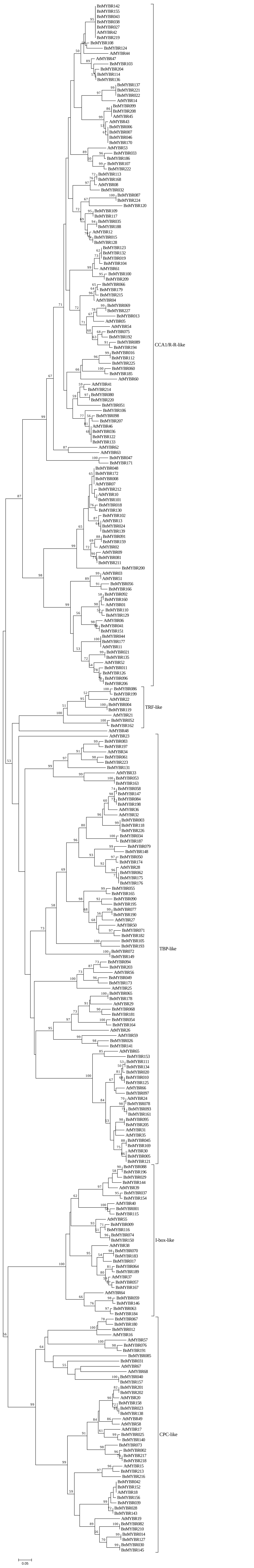

Figure S2. Phylogenetic tree of the MYB-related proteins (MYBRs) of *Brassica napus* and *Arabidopsis thaliana*. The unrooted tree was constructed using the neighbor-joining method based on the alignment of the MYB-like domains of the 227 *B. napus* and 68 *A. thaliana* MYBR proteins. Bootstrap values ≥ 50 (in percentage) are indicated along the branches. The MYBR proteins are clustered into five major subfamilies (designated as CCA1/R-R-like, I-box-like, CPC-like, TRF-like and TBP-like). The scale shows the amount of amino acid substitutions per site.
