# Supplementary material for: MYB Superfamily in Brassica napus: Evidence for Hormone-Mediated Expression Profiles, Large Expansion, and Functions in Root Hair Development
Source: Biomolecules. 2020 Jun 7;10(6):875. doi: 10.3390/biom10060875 (PMC7356979; doi:10.3390/biom10060875)
Supplement: Supplementary file 1 [file biomolecules-10-00875-s001.zip › Supplementary Materials/Figure S4.pdf]

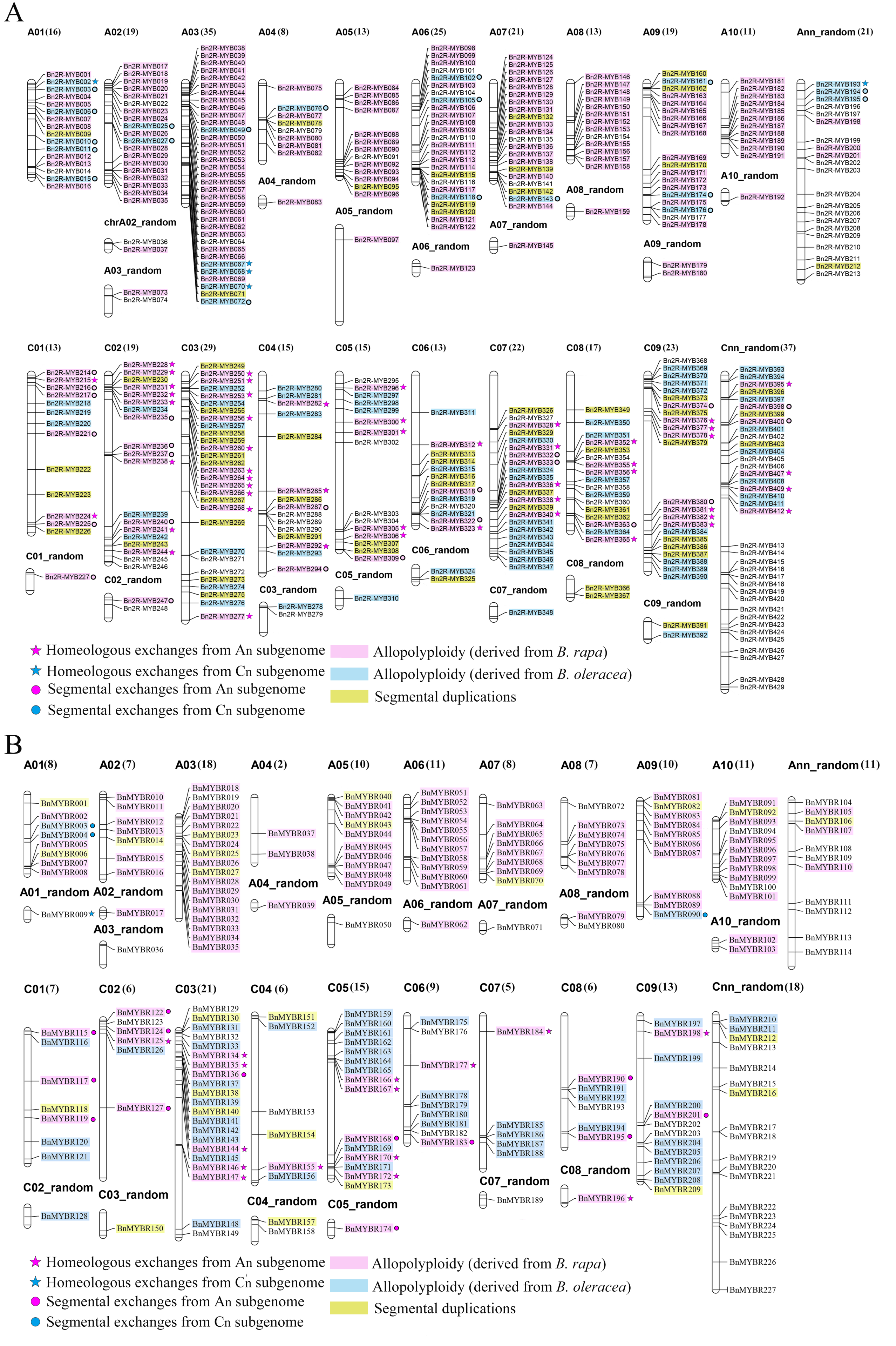

Figure S4. Physical positions and duplications of 429 *Bn2R-MYB* and 227 *BnMYBR* genes in 19 *Brassica napus* chromosomes. The chromosome numbers are indicated above each chromosome, and the numbers in the bracket indicate the *MYB* genes on each chromosome. The scale is in megabases (Mb). (A) Physical positions and duplication events of 429 *Bn2R-MYB* genes in 19 *B. napus* chromosomes. (B) Physical positions and duplication events of 227 *BnMYBR* genes in 19 *B. napus* chromosomes. Genes inherited from *B. rapa* or *B. oleracea* genomes are highlighted in pink and blue backgrounds, respectively. Genes originated from segmental duplication events are highlighted in yellow background. Genes derived from homeologous exchanges are marked with stars. Genes originated from segmental exchanges are indicated with dots.
