# Supplementary figures and images for: MYB Superfamily in Brassica napus: Evidence for Hormone-Mediated Expression Profiles, Large Expansion, and Functions in Root Hair Development
Source: Biomolecules. 2020 Jun 7;10(6):875. doi: 10.3390/biom10060875 (PMC7356979; doi:10.3390/biom10060875)

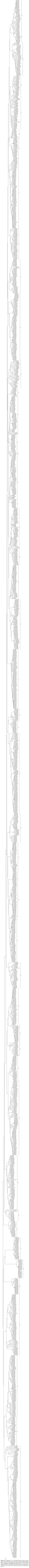

Supplement: Supplementary file 1 [file biomolecules-10-00875-s001.zip › Supplementary Materials/Figure S3.pdf]
